# Challenges of developing a digital scribe to reduce clinical documentation burden

**DOI:** 10.1038/s41746-019-0190-1

**Published:** 2019-11-22

**Authors:** Juan C. Quiroz, Liliana Laranjo, Ahmet Baki Kocaballi, Shlomo Berkovsky, Dana Rezazadegan, Enrico Coiera

**Affiliations:** Australian Institute of Health Innovation, Macquarie University, Sydney, Australia

**Keywords:** Health services, Software, Computational science, Information technology

## Abstract

Clinicians spend a large amount of time on clinical documentation of patient encounters, often impacting quality of care and clinician satisfaction, and causing physician burnout. Advances in artificial intelligence (AI) and machine learning (ML) open the possibility of automating clinical documentation with digital scribes, using speech recognition to eliminate manual documentation by clinicians or medical scribes. However, developing a digital scribe is fraught with problems due to the complex nature of clinical environments and clinical conversations. This paper identifies and discusses major challenges associated with developing automated speech-based documentation in clinical settings: recording high-quality audio, converting audio to transcripts using speech recognition, inducing topic structure from conversation data, extracting medical concepts, generating clinically meaningful summaries of conversations, and obtaining clinical data for AI and ML algorithms.

## Introduction

Clinical documentation is found to be associated with clinician burnout,^1^ increased cognitive load,^2^ information loss,^3^ and distractions.^4^ Ideally, clinical documentation would be an automated process, with only the minimally necessary input from humans. A digital scribe is an automated clinical documentation system able to capture the clinician–patient conversation and then generate the documentation for the encounter, like the function performed by human medical scribes.^5–9^ In theory, a digital scribe would enable a clinician to fully engage with a patient, maintain eye contact, and eliminate the need to split attention by turning to a computer to manually document the encounter. Reducing the time and effort invested by clinicians in the documentation process also has the potential to increase productivity, decrease clinician burnout, and improve the clinician–patient relationship, leading to higher quality and patient-centered care.^1^

Interest in digital scribes has increased rapidly. Along with academic research into digital scribes, a growing number of companies are also playing in the digital scribe space, including Microsoft, Google, EMR.AI, Suki, Robin Healthcare, DeepScribe, Tenor.ai, Saykara, Sopris Health, Carevoice, Notable, and Kiroku. Digital scribes can also be referred to as autoscribes, automated scribes, virtual medical scribes, artificial intelligence (AI) powered medical notes, speech recognition-assisted documentation, and smart medical assistants.^6,7^

**Fig. 1:**
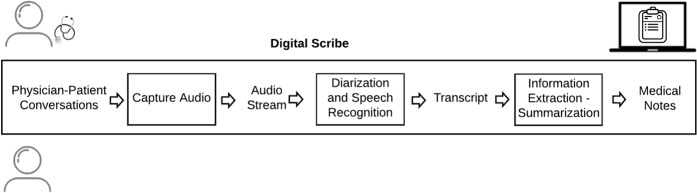
Digital scribe pipeline. A digital scribe acquires the audio of the clinician–patient conversation, performs automatic speech recognition to generate the conversation transcript, extracts information from the transcript, summarizes the information, and generates medical notes in the electronic health record (EHR) associated with the clinician–patient encounter. Speech recognition, information extraction, and summarization rely on AI and ML models that require large volumes of data for training and evaluation.

To generate medical notes for the clinician–patient encounter, a digital scribe must be able to: (1) record the clinician–patient conversation, (2) convert the audio to text, and (3) extract salient information from the text and summarize the information (Fig. 1). The implementation of a digital scribe consists of a pipeline of speech-processing and natural language processing (NLP) modules.^7^ Recently, advances in AI, machine learning (ML), NLP, natural language understanding, and automatic speech recognition (ASR), have raised the prospect of deploying effective and reliable digital scribes in clinical practice.

To date, research effort has focused on solving foundational problems in the development of a digital scribe, including ASR of medical conversations,^10,11^ automatically populating the review of symptoms discussed in a medical encounter,^12^ extracting symptoms from medical conversations,^13,14^ and generating medical reports from dictations.^15,16^ While these developments are promising, several challenges hinder the implementation of a fully functioning digital scribe and its evaluation in a clinical environment. This paper will discuss the major challenges, with a summary presented in Table 1.

**Table 1:** The challenges associated with the various tasks a digital scribe must perform.

Task	Challenge
Recording audio	• High ambient noise
	• Microphone fidelity
	• Multiple speakers
	• Microphone positioning relative to clinician and patient
Automatic speech recognition	• Varying audio quality
	• High ambient noise
	• Multiple speakers
	• Disfluencies, false starts, interruptions, non-lexical pauses
	• Complexity of medical vocabulary
	• Variable speaker volume due to distance to microphone and relative positioning
	• Differentiating multiple speakers in the audio (speaker diarization)
Topic segmentation	• Unstructured conversations
	• Non-linear progression of topics during a medical conversation
Medical concept extraction	• Noisy output of programs mapping text to UMLS
	• Tuning of parameters of tools used to map text to UMLS
	• Contextual inference (understanding the appropriate meaning of a word or phrase given the context)
	• Phenomena in spontaneous speech such as zero anaphora, thinking aloud, topic drift
Summarization	• Summarization of non-verbal unstructured communication
	• Integrating medical knowledge to identify relevant information
	• Contextual inference
	• Resolving conflicting information from the patient
	• Updating hypotheses as the patient discloses more information
	• Generating summaries to train a summarization ML model
Data collection	• Clinician and patient privacy concerns
	• Costly data collection and labeling
	• Patient consent to be audio recorded and use the data for research purposes
	• De-identification and anonymization of data
	• Expensive datasets
	• Data held privately as an intellectual property asset
	• Clinician reluctance to be recorded due to fear of legal liabilities and extra workload

## Challenge 1: audio recording and speech recognition

The first step for a digital scribe is recording the audio of a clinician–patient conversation. High-quality audio minimizes errors across the processing pipeline of the digital scribe. A recent study found that the word error rate of simulated medical conversations with commercial ASR engines was 35% or higher.^17^ These are best-case scenario results for current ASR technologies, as the recordings were made in a controlled environment, under near-ideal acoustic conditions, with speakers simulating a medical conversation while sitting in front of a microphone.

A recording made in a real clinical setting is likely to include noise and other environmental conditions that negatively affect ASR.^18,19^ The position of the recording device also has a strong impact on the captured audio.^18,20^ The clinician and the patient are unlikely to face the microphone during the consultation, as the sitting arrangement and physical examinations will affect their positions in relation to the recording device. This in turn affects the clarity and volume of the recorded audio.^17^ Having multiple speakers participating in the conversation and differentiating them in the audio (speaker diarization) also adds a level of complexity and potential errors to ASR.^7^ Recent work has shown the use of a recurrent neural network transducer significantly lowered diarization errors for audio recordings of clinical conversations between physicians and patients.^20^

Even with ideal recording equipment, ASR of conversational speech is more vulnerable to errors. Spontaneous, conversational speech is not linguistically well-formed.^21^ Conversations typically include disfluencies, such as interleaved false starts (e.g. “I’ll get, let me print this for you”), extraneous filler words (e.g. “ok”, “yeah”, “so”), non-lexical filled pauses (e.g. “umm”, “err”, “uh”), repetitions, interruptions, and talking over each other.^22,23^ Medical conversations have different statistical properties than medical dictations, meaning that ASR trained with dictations is likely to underperform with medical conversations.^7^ After conversion from speech to text, NLP techniques that perform well on grammatically correct sentences break down with conversational speech because of the lack of punctuation and sentence boundaries, grammatical differences between spoken and written language, and lack of structure.^7,24,25^

## Challenge 2: structuring clinician–patient conversations

**Fig. 2:**
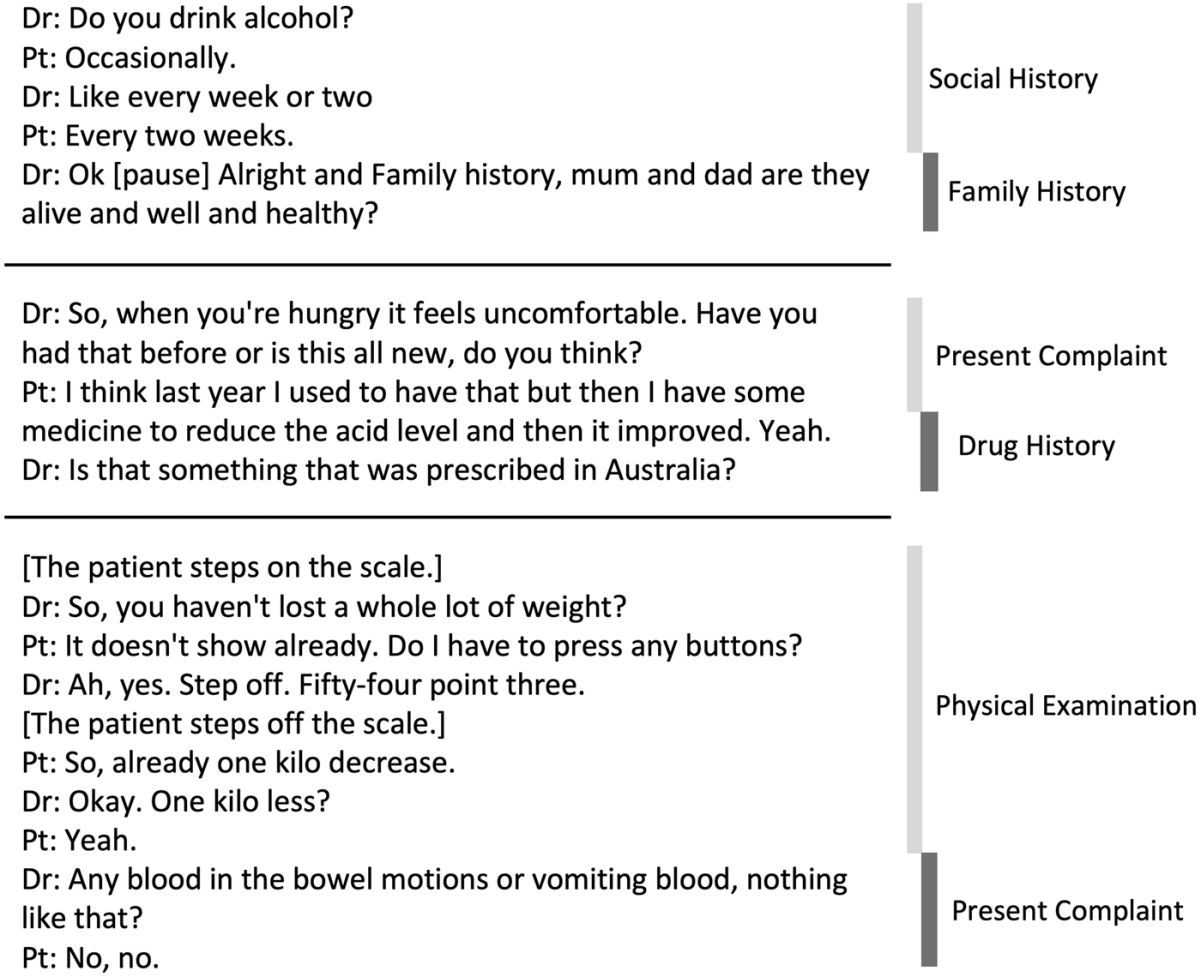
Three examples of transitions of clinician–patient conversations lacking clear boundaries and structure. Medical conversation fragments are on the left and the respective topics are on the right. Medical conversations do not appear to follow a classic linear model of defined information seeking activities. The nonlinearity of activities requires digital scribes to link disparate information fragments, merge their content, and abstract coherent information summaries.

ASR produces a transcript of the clinician–patient conversation that lacks clear boundaries and structure due to the unconstrained nature of conversations.^22^ That is, the content from one speaker turn to the next may be drastically different (consider example conversations in Fig. 2). One solution is to identify the category of each speaker turn (utterance), allowing for topic blocks to be identified in the transcripts (topic segmentation).^22,26^ Targeted information extraction and summarization can then be applied to the identified topics.^7,22^ The topics can be based on pre-determined categories^22^ or the components of a traditional medical encounter (chief complaint, family history, social history).^27^ However, clinical encounters do not necessarily follow a linear order of their components,^27,28^ which exacerbates summarization or information extraction.^22^

Knowing the current topic or medical activity during a consultation reduces the complexity of information extraction and summarization. For example, when discussing allergies, the doctor’s intent will include identifying the cause of the allergy (a particular substance or medication) and the body’s reaction. Information extraction for allergies can thus focus on identifying substances, medications, or food items as the cause of the allergy, and body parts and body reactions as the response to the allergen. In addition, topic identification can help single out information that can be ignored for documentation purposes, reducing the likelihood of including false positives or irrelevant information as part of the generated medical notes. For instance, if the clinician is explaining a condition to the patient, this segment of the conversation does not need to be summarized and stored in the electronic health record (EHR), even though it might contain a high proportion of medical terms.

## Challenge 3: information extraction in clinical conversations

Large-scale semantic taxonomies, such as the Unified Medical Language System (UMLS), allow for the identification of medical terminology in text. Existing tools, such as MetaMap and cTAKES, provide programmatic means for mapping text to concepts in the UMLS.^29^ However, UMLS was designed for written text, not for spoken medical conversations. The differences in (1) spoken vs. written language and (2) lay vs. expert terminology, cause inaccuracies and word mismatching when using existing tools for medical language processing from medical conversations.^30^ Tools like MetaMap must also have their parameters tuned, as using them with default settings may result in the extraction of irrelevant terms. With MetaMap’s default settings, the phrase “I am feeling fine” would result in “I” mapped to “blood group antibody I”, “feeling” mapped to “emotions”, and “fine” mapped to “qualitative concept” or “legal fine”. Therefore, additional steps must be taken to identify semantic types and groups to control the way text is mapped to medical concepts^29^ or develop rules to filter irrelevant terms, which depending on the text can be a time-consuming trial and error process.

A clinician–patient conversation is guided by clinicians’ emergent needs to obtain information about the patient’s condition. As a result, the information to be summarized is scattered throughout the dialog, requiring piecing together information from multiple utterances. Alternatively, several bits of information may be communicated in a single utterance. Research on machine comprehension of written passages cannot be directly transferred to spoken conversations due to common phenomena in spontaneous speech, such as zero anaphora (using an expression whose interpretation depends upon a prior expression), thinking aloud, and topic drift.^14^ In addition, conversations do not fit a command-like structure, which makes it difficult to perform intent recognition—identifying a user’s intent from an utterance^31^—and to apply NLP techniques.^19^ Finally, the large and complex medical vocabulary and the nature of conversations complicates contextual inference (understanding the appropriate meaning of a word or phrase given the context of nearby phrases or topic of the segment of the conversation), which is an integral part of making sense of the conversation.

## Challenge 4: conversation summarization

Generating a medical summary from a clinician–patient conversation can be cast as a supervised learning task,^32^ where an ML algorithm is trained with a large set of past medical conversation transcripts along with the gold standard summary associated with each conversation.^7,33^ The input to the summarization model would be a clinician–patient transcript and the output would be an appropriate summary.^34,35^ However, obtaining the gold standard summary of each conversation is costly because of the medical expertize required to complete the task^14^ and the high variability in clinician notes’ content, style, organization, and quality.^36^ Even if unsupervised learning is used to generate a summary, not requiring labels for the training data,^37,38^ a set of gold standard summaries would still be needed to evaluate the quality of the summarization.

To generate effective medical notes, the summarization may need to draw on medical knowledge and capture nonverbal information during a consultation. Medical notes include the most important points of the medical conversation, but also reflect specific information collected by the doctor by querying, listening, observing, physically examining the patient, and by drawing conclusions (some of which may never be communicated verbally). All these details may not be captured during a conversation, unless the clinician explicitly vocalizes what they are observing, experiencing, or thinking. Some changes in clinicians’ workflow or practices might be required to capture this information. For example, clinicians may need to vocalize their observations during physical examination. However, this may force clinicians to vocalize things that they may not want to tell the patients. Interaction design of such situations requires delicate resolutions. Future research should also focus on methods of integrating medical knowledge and nonverbal information as an input to ML or AI summarization models.

During a clinical encounter, it is common for a clinician to change their assessments or revise some observations. This will be difficult to differentiate by an automatic summarization model, as it would require sophisticated natural language understanding. A possible solution is to make clinicians responsible for editing and resolving conflicting information in the generated summary. Nevertheless, clinicians will only be convinced of embracing digital scribes if they believe that any reviewing and revising of summaries will be less time consuming than writing a summary from scratch.

## Challenge 5: lack of clinical data

Large scale public datasets have helped advance ML research by (1) providing researchers with the data at the scale necessary for building ML models and (2) facilitating research replication and benchmarks for comparing research. However, obtaining and sharing medical data presents a major obstacle due to privacy issues and the sensitive nature of the data.^13,14,39^ In some cases, government regulations may limit the sharing of data across global institutions and research teams. In other cases, the data is monetized.^40^ As a result, rich and accurate clinical data has become one of the most valuable intellectual property assets for industry and academia.

A new research team interested in collecting data, at the volumes needed to apply deep learning,^32,38,41^ would have to invest resources in buying or collecting the data at hospitals and clinics. Recent work describes the use of large volumes of data of medical conversations,^10,12,20^ but these are typically proprietary data repositories and not shared, in part due to the business and research advantages that access to such data offers and privacy limitations of sharing the data. Other work argued that lack of a publicly available corpus led the researchers to develop their own corpus of 3000 conversations annotated by medical scribes,^13^ a costly investment. Crowd-sourcing may be used to annotate large quantities of data from other domains, but it is less suitable for healthcare scenarios because of the need for domain knowledge to guarantee data quality.^14,42^ In general, digital scribe research is hindered by (1) limited well-annotated large-scale data for modeling human–human spoken dialogs and (2) even scarcer conversation data in healthcare due to privacy issues.^14^

There exist medical datasets for other ML tasks which are publicly available and exemplify how to share anonymized medical data for advancing medical research.^43–45^ A dataset of medical conversations along with the corresponding summaries would allow far-reaching advances in the digital scribe and clinical documentation space. Weak supervision has the potential to maximize the use of unlabeled medical data which is costly to annotate.^42^ Data trusts have also been proposed as a way of sharing medical data for research while giving users power over how their data is used.^46^ It remains to be seen how the implementation of data trusts affects the advancement of medical and AI research.

## Discussion: clinical practice implications

Along with a body of work advocating the use of AI and ML for automating clinical documentation,^6,8,47^ there are also arguments against this.^9^ The main concern raised is that manual documentation allows clinicians to structure their thoughts, think critically, reflect, and practice medicine effectively, such that removing it would adversely affect the way clinicians practice medicine.^9^ Current advocates of replacing the entire documentation process with AI also tend to overlook the complexities of healthcare sociotechnical systems.^9^ The evaluation of these systems in clinical environments must include an assessment of how they affect quality of care, patient satisfaction, clinician efficiency, documentation time, and organizational dynamics within a clinic. Research into unintended consequences of digital scribes need not wait until a fully functioning digital scribe prototype has been developed. These issues should be investigated through participatory workshop sessions with clinicians, patients, and other relevant stakeholders to inform the design of these systems.

Rather than replacement of clinicians as depicted in many dystopic AI futures, the goal of digital scribes is the formation of a “human–AI symbiosis” that augments the clinician–patient experience and improves quality of care.^6,8,9^ Digital scribes could well transform clinician–patient communication, bringing the focus back to the patient and clinical reasoning. The more seamless the digital scribe solution, the greater the support for the clinician engagement with patients. Any digital scribe solution that requires ongoing input and supervision throughout the consultation will (1) distract clinicians from patients and (2) replace the distractions and disruptions of using an EHR with those of a digital scribe. If the integration of a digital scribe comes at the expense of some standardization of clinical practice, this may still be worth it if it frees clinician time and improves the clinician–patient relationship. Standardization of some aspects of clinical encounters may also improve the patient understanding of clinical encounters.

## Conclusion

This paper presented several challenges to developing digital scribes. Future research should explore solutions to these pressing challenges, so that development and implemention of digital scribes may be advanced. Due to the complexity of each task, we posit that research may reap the greatest benefits by focusing on solving the challenges individually, as opposed to seeking to build a holistic solution. Adapting current ASR and NLP solutions to spontaneous, medical conversations needs to be a major research focus. In particular, conversational clinical data, transcripts, and summaries are needed to apply the recent advances in ML and AI to digital scribe development.

Collecting sufficient data at the scale needed for AI and ML algorithms could alone take years to complete. The currently closed environment for sharing sensitive data means that the few research teams with access to data are the only ones that can make advances, further slowing progress by impeding open science. Collective efforts must be made to make clinical data available for AI researchers to advance automated clinical documentation, while also protecting the data from misuse with ethical considerations in place.
